# Supplementary material for: Optomechanical time-domain reflectometry
Source: Nat Commun. 2018 Jul 31;9:2991. doi: 10.1038/s41467-018-05404-0 (PMC6068157; doi:10.1038/s41467-018-05404-0)
Supplement: Supplementary file 1 — Supplementary Information [file 41467_2018_5404_MOESM1_ESM.pdf]

Supplementary Information: Opto-Mechanical Time-Domain Reflectometry

Gil Bashan, Hilel Hagai Diamandi, Yosef London, Eyal Preter, and Avi Zadok

Supplementary Note 1:

Consider two co-polarized and continuous optical fields $\mathbf{E}_{1,2}$ that co-propagate in the positive z direction of an optical fibre:

$$\mathbf{E}_{1,2}(r,z,t) = A_{1,2}(z)E_\mathrm{T}(r)\exp\left(\mathrm{j}k_{1,2}z - \mathrm{j}\omega_{1,2}t\right)\mathbf{e} + c.c. \tag{1}$$

Here r and z are the radial and axial coordinates, respectively, and t stands for time. The optical frequencies of the two fields are $\omega_{1,2} = \omega_0 \pm \frac{1}{2}\Omega$, where ω_0 is a central optical frequency and Ω denotes spectral detuning. The value of Ω is chosen in the vicinity of the resonance frequency Ω_m of forward stimulated Brillouin scattering through a radial guided acoustic mode $\mathrm{R}_{0,m}$ of the fibre, where m is an integer. The wavenumbers of the two optical field components are $k_{1,2} = n\omega_{1,2}/c$, with n the effective modal index in the fibre and c the speed of light in vacuum. The normalized transverse profile of both fields is denoted by $E_\mathrm{T}(r)$. It is defined so that $2\pi\int_0^a \left|E_\mathrm{T}(r)\right|^2 r\mathrm{d}r = 1$, where a is the fibre cladding radius. $\mathbf{e}$ denotes a unit vector in the direction of the joint state of polarization of the two waves. The magnitudes of the two waves, $A_{1,2}(z)$, are affected by linear losses and may also vary along the fibre through coupling that is induced by the guided acoustic wave.

The overall optical power of the two fields comprises of DC terms, which do not contribute to guided acoustic waves stimulation, and the following beating term:

$$P_\mathrm{AC}(z,t) = 2nc\varepsilon_0 A_1(z)A_2^*(z)\exp\left(\mathrm{j}qz - \mathrm{j}\Omega t\right) + c.c., \tag{2}$$

where $q \equiv k_1 - k_2 = n\Omega/c$ and ε_0 is the vacuum permittivity. The interference component of the optical power gives rise to a radial electro-strictive driving force per unit volume[1,2]:

$$\mathbf{F}(r,z,t) = -\frac{\varepsilon_0}{2}(a_1 + 4a_2)E_\mathrm{T}(r)\frac{\mathrm{d}E_\mathrm{T}(r)}{\mathrm{d}r}A_1(z)A_2^*(z)\exp\left(\mathrm{j}qz - \mathrm{j}\Omega t\right)\mathbf{r} + c.c., \tag{3}$$

where $\mathbf{r}$ is a unit vector in the radial direction and $a_{1,2}$ are drawn from the photo-elastic tensor P of silica: $a_1 = -n^4\left(P_{11} - P_{12}\right)$, and $a_2 = -n^4 P_{12}$. The driving force may stimulate the oscillations of guided radial acoustic modes of the fibre $\mathrm{R}_{0,m}$. The axial phase velocity of these modes approaches infinity as $\Omega \to \Omega_m$. Hence there exists an acoustic frequency, very near cut-off, for which the axial phase velocity of the acoustic mode matches that of the optical mode[1,2]. The material displacement profile of modes $\mathrm{R}_{0,m}$ is entirely radial[1,2]:

$$\mathbf{U}^{(m)}\left(r,z,t\right) = B^{(m)}\left(z\right)u^{(m)}\left(r\right)\exp\left(\mathrm{j}qz - \mathrm{j}\Omega t\right)\mathbf{r} + c.c.. \tag{4}$$

In Supplementary Equation 4 the normalized transverse profile of the acoustic mode is given by:

$$u^{(m)}\left(r\right) = \frac{\mathrm{J}_1\left[\left(\Omega_m r\right)/v_\mathrm{d}\right]}{\sqrt{2\pi\int_0^a \left\{\mathrm{J}_1\left[\left(\Omega_m r\right)/v_\mathrm{d}\right]\right\}^2 r\mathrm{d}r}}. \tag{5}$$

Here v_d is the speed of longitudinal sound waves in silica, and J_1 denotes the first-order Bessel function of the first kind. The modal cut-off frequency is given by $\Omega_m = \left(v_\mathrm{d}\xi_m\right)/a$, where ξ_m is the mth-order solution to the boundary condition equation for zero traction force[1,2]:

$$\left(1 - \frac{v_\mathrm{s}^2}{v_\mathrm{d}^2}\right)\mathrm{J}_0\left(\xi\right) = \frac{v_\mathrm{s}^2}{v_\mathrm{d}^2}\mathrm{J}_2\left(\xi\right). \tag{6}$$

In Supplementary Equation 6 v_s is the speed of acoustic shear waves, and $\mathrm{J}_{0,2}$ denote the zero-order and second-order Bessel functions of the first kind, respectively. Lastly, the displacement magnitude $B^{(m)}\left(z\right)$ in Supplementary Equation 4 is determined by the magnitude of the electro-strictive driving force[1-9]:

$$B^{(m)}\left(z\right) = -\frac{\varepsilon_0}{\rho_0\Omega_m\left(z\right)\Gamma_m\left(z\right)}Q_{\mathrm{ES}}^{(m)}\frac{1}{\mathrm{j} - 2\Delta\Omega\left(z\right)/\Gamma_m\left(z\right)}A_1\left(z\right)A_2^*\left(z\right). \tag{7}$$

In Supplementary Equation 7 ρ_0 is the density of silica, $\Delta\Omega\left(z\right) \equiv \Omega - \Omega_m\left(z\right)$, and $\Gamma_m\left(z\right)$ is the spectral linewidth of the acoustic mode oscillations. Both resonance frequency and linewidth may vary with the mechanical impedance of the surrounding medium at position z. For an infinite medium of impedance that is much lower than that of silica, the effect may be

approximated in terms of modifications to the local modal linewidth[10]. The electro-strictive overlap integral $Q_{\mathrm{ES}}^{(m)}$ in Supplementary Equation 7 is defined as[9]:

$$Q_{\mathrm{ES}}^{(m)} \equiv 2\pi\left(a_1 + 4a_2\right)\int_0^a E_{\mathrm{T}}(r)\frac{\mathrm{d}E_{\mathrm{T}}(r)}{\mathrm{d}r}u^{(m)}(r)r\mathrm{d}r. \quad (8)$$

The acoustic wave oscillations are associated with perturbations to the dielectric constant tensor, denoted as $\delta\varepsilon^{(m)}(r,z)\exp(\mathrm{j}qz - \mathrm{j}\Omega t) + c.c.$. The elements of the dielectric perturbations tensor are determined by the transverse strain tensor that is associated with the material displacement $\mathbf{U}^{(m)}(r,z,t)$. They are provided in detail in earlier works[1-9]. For the purpose of this study, we require only the dielectric tensor perturbation that is averaged over the transverse profile of the optical mode: $\overline{\delta\varepsilon}^{(m)}(z) \equiv 2\pi\int_0^a \delta\varepsilon^{(m)}(r,z)\left|E_{\mathrm{T}}(r)\right|^2 r\mathrm{d}r$. For radial acoustic modes $\mathrm{R}_{0,m}$, the spatially-averaged dielectric perturbation is proportional to the unit tensor I: $\overline{\delta\varepsilon}^{(m)}(z) = \overline{\Delta\varepsilon}^{(m)}(z)I$, with a scalar value:

$$\overline{\Delta\varepsilon}^{(m)}(z) = Q_{\mathrm{PE}}^{(m)}B^{(m)}(z). \quad (9)$$

In Supplementary Equation 9 we have defined the photo-elastic overlap integral[9]:

$$Q_{\mathrm{PE}}^{(m)} \equiv 2\pi\left(\frac{a_1}{2} + a_2\right)\int_0^a \left[\frac{\mathrm{d}u^{(m)}(r)}{\mathrm{d}r} + \frac{u^{(m)}(r)}{r}\right]\left|E_{\mathrm{T}}(r)\right|^2 r\mathrm{d}r. \quad (10)$$

The acoustic perturbation to the dielectric tensor induces a nonlinear polarization vector:

$$\mathbf{P}_{\mathrm{NL}}^{(m)}(r,z,t) = \varepsilon_0\left[\delta\varepsilon^{(m)}(r,z)\exp(\mathrm{j}qz - \mathrm{j}\Omega t) + c.c.\right]\times\left[\mathbf{E}_1(r,z,t) + \mathbf{E}_2(r,z,t)\right], \quad (11)$$

The nonlinear polarization includes terms at the optical frequencies $\omega_{1,2}$:

$$\mathbf{P}_{\mathrm{NL},1}^{(m)}(r,z,t) = \varepsilon_0 A_2(z)\exp(\mathrm{j}k_1 z - \mathrm{j}\omega_1 t)E_{\mathrm{T}}(r)\delta\varepsilon^{(m)}(r,z)\mathbf{e} + c.c., \quad (12)$$

$$\mathbf{P}_{\mathrm{NL},2}^{(m)}(r,z,t) = \varepsilon_0 A_1(z)\exp(\mathrm{j}k_2 z - \mathrm{j}\omega_2 t)E_{\mathrm{T}}(r)\left[\delta\varepsilon^{(m)}(r,z)\right]^*\mathbf{e} + c.c. \quad (13)$$

The nonlinear polarization vector also includes pairs of terms at higher-order sideband frequencies $\omega_0 \pm \frac{3}{2}\Omega$. Higher-order Stokes and anti-Stokes field components may be amplified through opto-mechanics and Kerr nonlinearity[11]. However we do not address these terms in this work. The modulation of the incident optical waves is adjusted so that the input fields magnitudes at the frequencies of higher-order sidebands are very low. Subject to this condition, and for the lengths of fibres used in this work, our experiments show that higher-order sidebands remain sufficiently weak.

The expressions of Supplementary Equations 12 and 13 may be used to formulate the nonlinear wave equations for the propagation of the optical fields at frequencies $\omega_{1,2}$. For $\mathbf{E}_1$ at frequency ω_1 we obtain[12]:

$$\nabla^2\left[A_1(z)\exp(\mathrm{j}k_1 z)E_\mathrm{T}(r)\mathbf{e}\right]+n_\mathrm{f}^2(r)\frac{\omega_1^2}{c^2}\left[A_1(z)\exp(\mathrm{j}k_1 z)E_\mathrm{T}(r)\mathbf{e}\right] = -\frac{\omega_1^2}{\varepsilon_0 c^2}\varepsilon_0 A_2(z)\exp(\mathrm{j}k_1 z)E_\mathrm{T}(r)\delta\varepsilon^{(m)}(r,z)\mathbf{e}. \tag{14}$$

Here $n_\mathrm{f}(r)$ is the transverse profile of the refractive index of the unperturbed fibre. Since $E_\mathrm{T}(r)\exp(\mathrm{j}k_1 z)$ is a solution of the linear wave equation in the fibre, all terms on the left-hand side which do not involve z derivatives of $A_1(z)$ cancel out. In addition, we may invoke the slowly-varying envelope approximation and neglect the term that is proportional to $\mathrm{d}^2 A_1(z)/\mathrm{d}z^2$. The nonlinear wave equation then becomes[12]:

$$2\mathrm{j}k_1\frac{\mathrm{d}A_1(z)}{\mathrm{d}z}E_\mathrm{T}(r)\mathbf{e} = -\frac{\omega_1^2}{c^2}A_2(z)E_\mathrm{T}(r)\delta\varepsilon^{(m)}(r,z)\mathbf{e}. \tag{15}$$

Multiplying both sides by $E_\mathrm{T}^*(r)$, integrating over the transverse cross-section and substituting Supplementary Equation 9 on the right-hand side yield:

$$2\mathrm{j}k_1\frac{\mathrm{d}A_1(z)}{\mathrm{d}z} = -\frac{\omega_1^2}{c^2}A_2(z)\overline{\Delta\varepsilon}^{(m)}(z) = -\frac{\omega_1^2}{c^2}A_2(z)Q_\mathrm{PE}^{(m)}B^{(m)}(z). \tag{16}$$

Since the position-averaged dielectric perturbation tensor is proportional to the unit tensor, the vector notation in Supplementary Equation 16 could be dropped. The effect of radial acoustic modes on the optical fields is independent of polarization. Substituting Supplementary Equation 7, and with rearrangement of terms, the wave equation is brought to the following form:

$$\frac{\mathrm{d}A_1(z)}{\mathrm{dz}} = -\frac{\omega_1}{c}\frac{\varepsilon_0 Q_{\mathrm{ES}}^{(m)} Q_{\mathrm{PE}}^{(m)}}{2n\rho_0 \Omega_m(z)\Gamma_m(z)} \times \frac{1}{1+2\mathrm{j}\Delta\Omega(z)/\Gamma_m(z)} |A_2(z)|^2 A_1(z). \tag{17}$$

For simplicity, the optical frequency may be approximated as $\omega_1 \approx \omega_0$, leading to:

$$\frac{\mathrm{d}A_1(z)}{\mathrm{dz}} = -\frac{k_0 \varepsilon_0 Q_{\mathrm{ES}}^{(m)} Q_{\mathrm{PE}}^{(m)}}{2n\rho_0 \Omega_m(z)\Gamma_m(z)} \times \frac{1-\mathrm{j}\Delta\Omega(z)/\left[\frac{1}{2}\Gamma_m(z)\right]}{1+\left[\Delta\Omega(z)\right]^2/\left[\frac{1}{2}\Gamma_m(z)\right]^2} |A_2(z)|^2 A_1(z), \tag{18}$$

where $k_0 = \omega_0/c$ is the vacuum wavenumber at the central optical frequency. Similarly we obtain for the evolution of the magnitude of the second optical field $A_2(z)$:

$$\frac{\mathrm{d}A_2(z)}{\mathrm{dz}} = \frac{k_0 \varepsilon_0 Q_{\mathrm{ES}}^{(m)} Q_{\mathrm{PE}}^{(m)}}{2n\rho_0 \Omega_m(z)\Gamma_m(z)} \times \frac{1+\mathrm{j}\Delta\Omega(z)/\left[\frac{1}{2}\Gamma_m(z)\right]}{1+\left[\Delta\Omega(z)\right]^2/\left[\frac{1}{2}\Gamma_m(z)\right]^2} |A_1(z)|^2 A_2(z). \tag{19}$$

The coupled nonlinear wave equations may be converted to describe the evolution of the optical powers of the two fields $P_{1,2}(z) \equiv 2nc\varepsilon_0 |A_{1,2}(z)|^2$, using the relation $\mathrm{d}P_{1,2}/\mathrm{dz} = 2nc\varepsilon_0 \cdot 2\,\mathrm{Re}\left\{A_{1,2}^* \cdot \mathrm{d}A_{1,2}/\mathrm{dz}\right\}$:

$$\begin{aligned}\frac{\mathrm{d}P_1(z)}{\mathrm{dz}} &= -\frac{k_0 Q_{\mathrm{ES}}^{(m)} Q_{\mathrm{PE}}^{(m)}}{2n^2 c\rho_0 \Omega_m(z)\Gamma_m(z)} \times \frac{1}{1+\left[\Delta\Omega(z)\right]^2/\left[\frac{1}{2}\Gamma_m(z)\right]^2} P_1(z)P_2(z) \\ &= -2\gamma^{(m)}(\Omega, z)P_1(z)P_2(z),\end{aligned} \tag{20}$$

$$\begin{aligned}\frac{\mathrm{d}P_2(z)}{\mathrm{dz}} &= \frac{k_0 Q_{\mathrm{ES}}^{(m)} Q_{\mathrm{PE}}^{(m)}}{2n^2 c\rho_0 \Omega_m(z)\Gamma_m(z)} \times \frac{1}{1+\left[\Delta\Omega(z)\right]^2/\left[\frac{1}{2}\Gamma_m(z)\right]^2} P_1(z)P_2(z) \\ &= 2\gamma^{(m)}(\Omega, z)P_1(z)P_2(z).\end{aligned} \tag{21}$$

Supplementary Equations 20 and 21 show that the stimulation of the acoustic wave leads to the amplification of the optical power of the wave component at frequency $\omega_0 - \frac{1}{2}\Omega$, and to the attenuation of the optical power of the tone at frequency $\omega_0 + \frac{1}{2}\Omega$. Here we have used previous definitions for the nonlinear coefficient associated with opto-mechanical coupling[9,13], in units of $\mathrm{W}^{-1}\times\mathrm{km}^{-1}$:

$$\gamma^{(m)}(\Omega,z) \equiv \frac{k_0 Q_{\mathrm{ES}}^{(m)} Q_{\mathrm{PE}}^{(m)}}{4n^2 c\rho_0 \Omega_m(z)\Gamma_m(z)} \times \frac{1}{1+\left[\Delta\Omega(z)\right]^2 / \left[\frac{1}{2}\Gamma_m(z)\right]^2} = \gamma_0^{(m)}(z)\frac{1}{1+\left[\Delta\Omega(z)\right]^2 / \left[\frac{1}{2}\Gamma_m(z)\right]^2}, \tag{22}$$

where the maximum coefficient is obtained on resonance:

$$\gamma_0^{(m)}(z) \equiv \frac{k_0}{4n^2 c\rho_0}\frac{Q_{\mathrm{ES}}^{(m)} Q_{\mathrm{PE}}^{(m)}}{\Omega_m(z)\Gamma_m(z)}. \tag{23}$$

The separations between the resonance frequencies of scattering due to adjacent radial modes are larger than the linewidths: $\left|\Omega_{m\pm1}(z)-\Omega_m(z)\right| >> \Gamma_m(z)$ for all m and z, so that opto-mechanical coupling at a given Ω may take place through a single radial mode, at most. In standard single-mode fibre with standard dual-layer acrylate coating, the nonlinear coefficient is the largest for mode $\mathrm{R}_{0,7}$ [14]: $\gamma_0^{(7)}$ ~ 2.5 $\mathrm{W}^{-1}\times\mathrm{km}^{-1}$. This value is twice larger than the nonlinear coefficient of the Kerr effect in the same fibre. The resonance frequency for scattering by that mode is Ω_7 ~ 2π×320 MHz[1,14]. In principle, $P_{1,2}(z)$ may also be affected by a specific class of torsional-radial guided acoustic modes of the fibre, noted as $\mathrm{TR}_{2,m}$ [1,2]. However, the nonlinear coefficients associated with these modes are considerably weaker.

The nonlinear coefficients may vary with fibre position, since both the resonance frequency $\Omega_m(z)$ and the linewidth $\Gamma_m(z)$ depend on the exact cladding radius, strain, temperature, mechanical impedance of media outside the cladding etc. Supplementary Equations 20 and 21 are readily extended to include linear propagation losses with a coefficient α :

$$\frac{\mathrm{d}P_1(z)}{\mathrm{dz}} = -\alpha P_1(z) - 2\gamma^{(m)}(\Omega,z)P_1(z)P_2(z), \tag{24}$$

$$\frac{\mathrm{d}P_2(z)}{\mathrm{dz}} = -\alpha P_2(z) + 2\gamma^{(m)}(\Omega,z)P_1(z)P_2(z). \tag{25}$$

The coupled power equations are analogous to those of forward stimulated Raman scattering[15]. Due to this similarity, guided acoustic wave Brillouin scattering is often referred to as a Raman-like process[16]. For uniform fibres in which the nonlinear coefficient is independent of z, the pair of equations may be solved analytically[15]:

$$P_1(z) = P_1(0)\frac{1+M^{-1}}{1+M^{-1}\exp\left(g^{(m)}L_{\text{eff}}\right)}\exp(-\alpha z), \tag{26}$$

$$P_2(z) = P_2(0)\frac{1+M}{1+M\exp\left(-g^{(m)}L_{\text{eff}}\right)}\exp(-\alpha z). \tag{27}$$

Here $P_{1,2}(0)$ are the incident optical powers of the two tones, $M \equiv P_1(0)/P_2(0)$, L_{eff} is the fibre effective length, and $g^{(m)} \equiv 2\gamma^{(m)}(\Omega)P_1(0)\left(1+M^{-1}\right)$. If the fibre is non-uniform, Supplementary Equations 24 and 25 may be integrated numerically instead.

The local nonlinear coefficient of opto-mechanical coupling can be evaluated based on Supplementary Equations 24 and 25:

$$\gamma^{(m)}(\Omega,z) = \frac{1}{4P_1(z)P_2(z)}\left\{\frac{\mathrm{d}\left[P_2(z)-P_1(z)\right]}{\mathrm{d}z}+\alpha\left[P_2(z)-P_1(z)\right]\right\}. \tag{28}$$

The distributed sensing protocol proposed in this work reconstructs the local values of opto-mechanical coupling coefficients based on estimates of $P_{1,2}(z)$, which are obtained through measurements of incoherent Rayleigh backscatter (see Main Text). We therefore refer to the sensing principle as opto-mechanical time-domain reflectometry, or OM-TDR. Spatially-resolved information is recovered through modulating the amplitudes of the two input tones by a single pulse of duration τ. The above steady-state analysis remains valid, provided that $\tau > 1/\Gamma_m(z)$ for all z. The linear losses coefficient α may be pre-calibrated using standard optical time-domain reflectometry. Measurements are repeated for multiple values of frequency detuning Ω, to obtain a distributed mapping of forward stimulated Brillouin scattering gain spectra.

Supplementary References

1. Shelby, R. M., Levenson, M. D., and Bayer, P. W. Guided Acoustic-Wave Brillouin Scattering. *Phys. Rev. B*, **31**, 5244-5252 (1985).
2. Biryukov, A. S., Sukharev, M. E., and Dianov, E. M. Excitation of Sound Waves Upon Propagation of Laser Pulses in Optical Fibers. *IEEE J. Quant. Elect.*, **32**, 765-775 (2002).
3. Renninger, W. *et al*. Forward Brillouin scattering in hollow-core photonic bandgap fibers. *New J. Physics*, **18**, 025008 (2016).

4. Renninger, W., Behunin, R. O., and Rakich, P. T. Guided-wave Brillouin scattering in air. *Optica*, **3**, 1313-1319 (2016).
5. Godet, A. *et al*. Brillouin spectroscopy of optical microfibers and nanofibers. *Optica*, **4**, 1232-1238 (2017).
6. Beugnot, J.-C. *et al*. Brillouin light scattering from surface acoustic waves in a subwavelength-diameter optical fibre. *Nat. Commun*., **5**, 5242 (2014).
7. Florez, O. *et al*. Brillouin scattering self-cancellation. *Nat. Commun.*, **7**, 11759 (2016).
8. Beugnot, J.-C., Sylvestre, T., Maillotte, H., Mélin, G., V. Lande, V. Guided acoustic wave Brillouin scattering in photonic crystal fibers. *Opt. Lett*., **32**, 17–19 (2007).
9. Diamandi, H. H., London, Y., and Zadok, A. Opto-mechanical inter-core cross-talk in multi-core fibers. *Optica*, **4**, 289-297 (2017).
10. Antman, Y., Clain, A., London, Y., and Zadok, A. Optomechanical sensing of liquids outside standard fibers using forward stimulated Brillouin scattering. *Optica*, **3**, 510-516 (2016).
11. Wolff, C., Stiller, B., Eggleton, B. J., Steel, M. J., and Poulton, C. G. Cascaded forward Brillouin scattering to all Stokes orders. *New J. Physics*, **19**, 023021 (2017).
12. Boyd, R. W. Nonlinear Optics 3rd Edition, (Academic, 2008).
13. Butsch, A. *et al*. Optomechanical Nonlinearity in Dual-Nanoweb Structure Suspended Inside Capillary Fiber. *Phys. Rev. Lett*., **109**, 183904 (2012).
14. London, Y., Diamandi, H. H., and Zadok, A. Electro-opto-mechanical radio-frequency oscillator driven by guided acoustic waves in standard single-mode fiber. *Appl. Phys. Lett. Photon*., **2**, 041303 (2017).
15. Agrawal, G. P. Nonlinear Fiber Optics (Academic, 2007).
16. Kang, M. S., Nazarkin, A., Brenn, A., and Russell, P. St. J. Tightly trapped acoustic phonons in photonic crystal fibers as highly nonlinear artificial Raman oscillators. *Nat. Phys*., **5**, 276–280 (2009).
